# Brain Activity Foreshadows Stock Price Dynamics

**DOI:** 10.1523/JNEUROSCI.1727-20.2021

**Published:** 2021-04-07

**Authors:** Mirre Stallen, Nicholas Borg, Brian Knutson

**Affiliations:** Department of Psychology, Stanford University, Stanford, California 94305

**Keywords:** accumbens, choice, decision, financial, forecast, insula

## Abstract

Successful investing is challenging since stock prices are difficult to consistently forecast. Recent neuroimaging evidence suggests, however, that activity in brain regions associated with anticipatory affect may not only predict individual choice, but also forecast aggregate behavior out-of-sample. Thus, in two experiments, we specifically tested whether anticipatory affective brain activity in healthy humans could forecast aggregate changes in stock prices. Using functional magnetic resonance imaging, we found in a first experiment (*n* = 34, 6 females; 140 trials/subject) that nucleus accumbens activity forecast stock price direction, whereas anterior insula (AIns) activity forecast stock price inflections. In a second preregistered replication experiment (*n* = 39, 7 females) that included different subjects and stocks, AIns activity still forecast stock price inflections. Importantly, AIns activity forecast stock price movement even when choice behavior and conventional stock indicators did not (e.g., previous stock price movements), and classifier analysis indicated that forecasts based on brain activity should generalize to other markets. By demonstrating that AIns activity might serve as a leading indicator of stock price inflections, these findings imply that neural activity associated with anticipatory affect may extend to forecasting aggregate choice in dynamic and competitive environments such as stock markets.

**SIGNIFICANCE STATEMENT** Many try but fail to consistently forecast changes in stock prices. New evidence, however, suggests that anticipatory affective brain activity may not only predict individual choice, but also may forecast aggregate choice. Assuming that stock prices index collective choice, we tested whether brain activity sampled during the assessment of stock prices could forecast subsequent changes in the prices of those stocks. In two neuroimaging experiments, a combination of previous stock price movements and brain activity in a region implicated in processing uncertainty and arousal forecast next-day stock price changes—even when behavior did not. These findings challenge traditional assumptions of market efficiency by implying that neuroimaging data might reveal “hidden information” capable of foreshadowing stock price dynamics.

## Introduction

Although investors strive to forecast changes in stock prices, most fail to consistently do so. Accordingly, traditional finance theory implies that investors should not be able to reliably forecast stock prices (Fama, 1970), although behavioral finance researchers have identified exceptions (Farmer and Lo, 1999; Barberis and Thaler, 2003; Shiller, 2003; Hirshleifer, 2015). Forecasting stock prices might prove challenging for many reasons, including random variation in systematic preferences of investors, as well as arbitrage of naive investors' systematic preferences by more sophisticated investors (Camerer, 2003; Barberis, 2018).

Despite the challenge of translating individual predictions into aggregate forecasts, recent neuroimaging work suggests that some neural predictors of individual choice might further scale to forecast aggregate choice (Falk et al., 2012; Knutson and Genevsky, 2018). For instance, average group neural activity in laboratory samples has been used to forecast aggregate market responses to music clips (Berns and Moore, 2012), advertisements (Venkatraman et al., 2015), microloan appeals (Genevsky and Knutson, 2015), crowdfunding proposals (Genevsky et al., 2017), news summaries (Scholz et al., 2017), and video clips (Tong et al., 2020). In some cases, experimentally measured neural activity can even forecast aggregate choice better than stated preferences or behavioral choices. These collected findings imply that some neural processes occurring before individual choices may generalize to forecast others' choices, and may do so more robustly than other neural processes or even behavior (Knutson and Genevsky, 2018).

We sought to extend this “neuroforecasting” approach in a critical new direction by examining whether experimentally measured brain activity can forecast changes in stock prices. We specifically tested whether brain activity sampled from a group of individuals assessing and investing in stocks might reveal useful information about impending stock price changes. Forecasting stock price dynamics presents a significant new challenge, since stock prices reflect not only the aggregate choices of individuals (in which increased purchases drive prices up, while increased sales drive prices down), but also dynamic interactions and competition between individuals (De Martino et al., 2013). Understanding whether neural processes forecast stock price dynamics might yield insights into which neural mechanisms generalize across individuals to forecast aggregate choice in general, and further test whether brain activity extends to forecast aggregate behavior in dynamic and competitive environments like stock markets.

Building from the notion that anticipatory affect can precede and predict risky choice in individuals (Bechara et al., 1996; Loewenstein et al., 2001; Knutson and Greer, 2008), we hypothesized that sampled brain activity associated with positive aroused affect and approach behavior [i.e., Nucleus Accumbens (NAcc) activity] would forecast increased demand for stocks and associated price increases (i.e., price direction), but that brain activity associated with negative or generally aroused affect and avoidance behavior [i.e., anterior insula (AIns) activity] would instead forecast decreased or changing demand for stocks and associated price decreases or changes (i.e., price inflections; Paulus et al., 2003; Kuhnen and Knutson, 2005; Knutson and Huettel, 2015). Further, and consistent with a “partial scaling” account (Knutson and Genevsky, 2018), we hypothesized that activity in deeper brain regions associated with anticipatory affect might forecast aggregate choice—even when activity in more cortical regions associated with value integration [e.g., the medial prefrontal cortex (MPFC)] and subsequent choice behavior do not. We tested these hypotheses first in a neuroimaging experiment, and then examined the replicability and generalizability of those findings in a second preregistered neuroimaging experiment.

## Materials and Methods

### 

#### Experimental design

##### Subjects.

Forty-one healthy subjects were recruited and scanned for experiment 1 and 49 healthy subjects were recruited and scanned for (preregistered) experiment 2. The sample size for experiment 1 was based on a review of previous neuroforecasting research (Knutson and Genevsky, 2018). Exclusion criteria included typical magnetic resonance safety criteria (e.g., no metal in the body or fear of enclosed spaces), as well as history of psychotropic drug use, brain damage, alcoholism, substance use, or cardiac medications. For experiment 1, six subjects were excluded for excessive head motion during scanning (i.e., >4 mm of movement from one image volume acquisition to the next) and one subject was excluded because of incomplete data acquisition, leaving a total of 34 subjects for analysis (6 females; age range = 22–43 years; mean age = 29.1 years; SD = 5.35). For experiment 2, 7 subjects were excluded for excessive head motion during scanning and 3 subjects were excluded because of incomplete data acquisition, leaving a total of 39 subjects for analysis (7 females; age range = 18–47 years; mean age = 27.5 years; SD = 6.14). Most subjects were students at Stanford University, no expertise in financial investing was required, and subjects reported that they either did not invest at all or only invested in personal (not professional) accounts. Consistent with the sex imbalance typically observed in professional traders, more males than females volunteered.

Subjects received $20/h for participating, as well as the opportunity to keep any money they gained based on their performance in the asset pricing task (APT) and an unrelated subsequent financial decision-making task (not described here). Subjects earned an average of $10.40 (SD, $0.36) per stock in experiment 1 and $10.29 (SD, $0.41) per stock in experiment 2 (which included their $10.00 starting endowment for each stock). All procedures were conducted as approved by the Institutional Review Board on Medical Human Subjects of Stanford University.

##### Procedure.

After providing informed consent, subjects read the instructions and completed several practice trials for the experimental task of interest (i.e., the asset pricing task; described below) as well as practice trials for a subsequent and different financial decision-making task. In experiment 1, the second task was the behavioral investment allocation strategy task (Kuhnen and Knutson, 2005), and in experiment 2 the second task was a gambling task (Leong et al., 2016)—findings related to these tasks will be described elsewhere. Before and after scanning, subjects completed questionnaires assessing sociodemographic information and individual differences in affective experience and cognitive abilities (adapted from Knutson et al., 2011).

##### Asset pricing task

**Figure 1.**
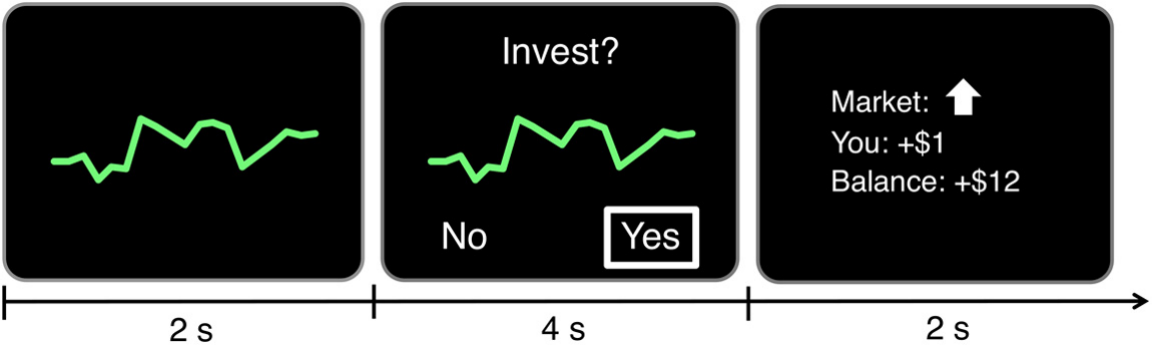
Asset pricing task trial structure. Trials included presentation of a stock trend line (2 s, left); choice to invest (4 s, middle), and outcome (2 s, right). A central fixation cross (2–6 s), was presented between trials (not depicted).

To assess brain activity related to stock price dynamics, we designed a novel APT suitable for use with functional magnetic resonance imaging (fMRI). The APT displays trend lines that sequentially and dynamically depict historical prices of real stocks. After each daily price update, subjects chose whether to either invest in the displayed stock or not (Fig. 1). Stock trend lines depicted daily closing prices and came from 14 different stocks selected from the S&P 500 index and extracted from online finance data (listed on https://finance.yahoo.com). For each experiment, we randomly selected a 30 d trading period in 2015 (October 28 to December 9, 2015, for experiment 1; March 4 to April 15, 2015 for experiment 2), which represented recent markets relative to the time when the experiments were conducted (i.e., in 2016). For experiment 1, 14 stocks were randomly selected from the S&P 500 index. For experiment 2, 14 stocks were pseudorandomly selected from the S&P 500 index to exclude stocks used in experiment 1, as well as to avoid incidental autocorrelation within and between stocks. Specifically, to select stocks for experiment 2, we estimated an ordinary least-squares regression model for each stock based on the stock prices of the selected 30 d trading period. Then, stocks were divided into six bins based on their slope (i.e., β value of the regression model was >0 or <0) and volatility (i.e., residual sum of squares of the regression model was either low, medium, or high). Next, two or three stocks were randomly selected from each of these bins to yield a random but stratified set of 14 stocks that varied in terms of slope and volatility. Stocks that were included in experiment 1 were excluded from selection in experiment 2. In both experiments, stock prices were converted to *z* scores to fit their trend lines on a common vertical value axis for display. Importantly, subjects were not informed about which stock identities or time periods were sampled.

During the task, subjects viewed sequentially updating trend lines corresponding to each of the 14 stocks (10 trials/stock). Stock price trend lines were displayed using a “rolling window” format, such that each of the 10 updates showed a trend line of 20 previous price updates along with the most recent update at its end (i.e., on the right). For each stock, subjects began with a $10.00 endowment, after which they made 10 consecutive investment choices after the displayed trend line was updated. Stocks were thus presented in 10-trial blocks, in one of two pseudorandomized orders.

During each task trial, subjects initially saw a trend line reflecting the price history of the stock over 20 previous updates (for 2 s), followed by a choice prompt to indicate whether they wanted to either invest ($1.00) in that stock or not via button press (i.e., “Yes” or “No,” laterally spatially counterbalanced; 4 s). If subjects invested and the stock price then increased, their balance increased by $1.00 but if subjects invested and the stock price then decreased, their balance decreased by $1.00. Thus, given an approximately even probability of stock price increasing or decreasing, the overall expected value of either investing or not investing on any trial was approximately $0.00. After choosing whether to invest or not, a feedback screen revealed whether the stock price had in fact increased or decreased, along with the amount of money the subject had gained or lost as a consequence of their choice and their cumulative overall balance (2 s). Finally, subjects visually fixated on a centrally presented cross (2–6 s) while awaiting the start of the next trial (Fig. 1).

At the end of each 10-trial block, subjects were instructed to imagine that they had an opportunity to invest in more shares of that stock as a trader, and to indicate their choice to buy, sell, or hold (i.e., neither to buy nor to sell) the stock with a button press (6 s). Subjects then rated their confidence in their choice (i.e., by selecting one of 0–25%, 26–50%, 51–75%, and 76–100% response options; 6 s). These final choices and confidence ratings are not further analyzed here since subjects' trial-to-trial choices to invest provided the critical behavioral variables of interest for the current forecasting analyses. The total amount of money gained (or lost) during each block was added to (or subtracted from) subjects' initial $10.00 endowment. At the end of each experiment, 4 of the 14 blocks were randomly selected, and the average payment over these four blocks was added to subjects' hourly base payment. Thus, both experiments used no deception and were fully incentive compatible. The task was divided into two scanning runs including seven stocks per run with trend lines of 10 price updates (trials) each, totaling 140 trials that lasted 32 min.

#### Statistical analysis

##### fMRI acquisition and analysis.

Images were acquired with a 3.0 T MRI scanner (General Electric) using a 32-channel head coil. Forty-six 2.9-mm-thick slices (in-plane resolution, 2.9 mm; isotropic, no gap, interleaved acquisition) extended axially from the midpons superiorly to the crown of the skull to provide whole-brain coverage. Whole-brain functional scans were acquired with a T2*-weighted gradient-echo pulse sequence (repetition time, 2 s; echo time, 25 ms; flip angle, 77°). High-resolution structural scans were acquired after functional scans with a T1-weighted pulse sequence (repetition time, 7.2 ms; echo time, 2.8 ms; flip angle, 12°) to facilitate their localization and coregistration.

Analyses of fMRI data were conducted using Analysis of Functional Neural Images (AFNI) software, version AFNI_18.0.25 (Cox, 1996). For preprocessing, voxel time series were concatenated across runs; sinc interpolated to correct for nonsimultaneous slice acquisition within each volume; motion corrected; spatially smoothed to minimize the effects of anatomic variability while retaining sufficient resolution to visualize structures of interest (4 mm full-width at half-maximum kernel); normalized to the percentage signal change with respect to the average of each voxel over the entire task; and high-pass filtered to omit frequencies with periods >90 s.

To extract brain data for testing the critical predictions, targeted analyses focused on data extracted from three predefined volumes of interest (VOIs) whose activity previously predicted individual choice in studies of financial risk-taking (Kuhnen and Knutson, 2005), as well as forecast market-level behavior (Knutson and Genevsky, 2018). These meta-analytically derived (Knutson and Greer, 2008) VOIs specifically centered on predefined bilateral foci (8-mm-diameter spheres) in the NAcc (Talairach focus: *x*, ±10; *y*, +12; *z*, −2), the AIns (Talairach focus: *x*, ±28; *y*, +18; *z*, –5), and the MPFC (Talairach focus: *x*, ±4; *y*, +45; *z*, 0). Activity time courses were first normalized over time within each voxel, and then averaged over voxels comprising each VOI. For forecasting analyses, brain activity was averaged that corresponded to the presentation of the stock price update, lagged for the hemodynamic response by 6 s (i.e., the fourth 2 s volume acquisition after trial onset) before being entered into models. Activity exceeding ≥4 SDs was omitted before analyses, in addition to trials in which stock prices remained stable across 2 d (four trials in experiment 1, and two trials in experiment 2) since they could not be classified as displaying a price increase or decrease.

To test whether neural activity could forecast stock price dynamics, logistic regression analyses that forecast next-day aggregate stock price movement then were conducted on data clustered by stock and averaged over subjects (i.e., 10 price updates per stock averaged over all subjects in the sample; all regression analyses were conducted using the lme4 package version 1.1–21 of the R statistical language; R Core Team, 2018). These models included fixed effects of the following: (1) stock indicators (Market model); (2) average choice to invest or not (Behavioral model); (3) neural activity averaged over VOIs (the NAcc, AIns, and MPFC) in response to presentation of stock price updates (Neural model); and (4) all of these components combined (Combined model). For the Market and Combined models, stock indicators included stock price movement on the previous day (i.e., price increase vs decrease), and the slope and volatility indicators of each updated trend line. To calculate slope and volatility indicators, we estimated an ordinary least-squares regression model for each updated trend line (10 updates/stock, so 10 regression models/stock). The slope and volatility indicators reflected, respectively, the β and residual sum of squares of each regression model that was estimated using the updated trend line presented on a given trial. For outcome variables, price direction indexed continuation (i.e., the price increased after increasing on the previous trial or decreased after decreasing on the previous trial), whereas price inflection indexed reversals (i.e., the price decreased after increasing on the previous trial or increased after decreasing on the previous trial). Likelihood ratio tests were used to test whether the Combined model performed significantly better or worse than the other models (using the lrtest function of the R lmtest package version 0.9–34).

To establish whether neural forecasts could generalize across markets, we trained a linear support-vector-machine classifier on the behavioral, neural, and stock indicator data from experiment 1 (or experiment 2), and tested whether this classifier could predict stock price movement of the stocks used in experiment 2 (or experiment 1) above chance (using the e1071 R package, version 1.7–2; R Core Team, 2018). Classifiers were trained on the Combined model as well as on a reduced model that only included anticipatory AIns activity, stock price movement on the previous trial, and their interaction. Since subsequent stock price movement was the outcome variable, data were downsampled to include 50% increases and 50% decreases of stock prices. Binomial tests then evaluated whether classifiers could forecast stock price movement out-of-sample above chance (i.e., 50%, consistent with the Efficient Market Hypothesis). To further verify whether classifiers could forecast stock prices, classifiers were additionally trained on randomized stock prices of experiment 1 (or experiment 2) and then tested on nonrandomized data of experiment 2 (or experiment 1), with the assumption that training on random data should produce a null result. Stock prices were randomized within each experiment 500 times to reduce estimation dependence on any particular randomized order. One-sample *t* tests were used to compare whether test accuracies of models trained on randomized stock prices significantly exceeded chance.

To verify task engagement and accurate selection of the predefined volumes of interest, two whole-brain analyses were conducted. A first whole-brain analysis contrasted individual brain activity in response to different outcomes. For this analysis, increased NAcc activity was expected in response to gains (i.e., price increases after choosing to invest) as well as to avoided loss outcomes (i.e., counterfactual price decreases after choosing not to invest; Kuhnen and Knutson, 2005; Lohrenz et al., 2007). Whole-brain regression models analyzing neural activity in response to outcomes included 15 regressors. Twelve regressors were not of interest [i.e., six regressors indexing residual motion, two that indexed activity associated with CSF and white matter intensity (Chang and Glover, 2009), and four that modeled each of the trial periods]. The following two orthogonal regressors of interest contrasted: (1) outcomes following investment choices (i.e., price increase and financial gain vs price decrease and financial loss after choices to invest; onset, feedback screen; duration, 2 s); and (2) outcomes following choices not to invest (i.e., price decrease or counterfactual gain vs price increase or counterfactual loss after choices not to invest; onset, feedback screen; duration, 2 s).

A second whole-brain analysis confirmed that average activity in predicted regions forecast next-day aggregate stock price movement. This model included 12 regressors that were not of interest, including regressors indexing the following: residual motion, regressors 1–6; activity associated with CSF and white matter intensity (Chang and Glover, 2009), regressors 7–8; each of the trial periods, regressors 9–12. Two orthogonal regressors of interest contrasted upcoming stock price, as follows: (1) direction (price increase vs decrease; onset, stimulus screen; duration, 4 s); and (2) inflection (i.e., price direction changes vs continuation; onset, stimulus screen; duration, 4 s). For both whole-brain analyses, all regressors of interest were convolved with a single γ-variate function modeling a canonical hemodynamic response function. Maps of *t* statistics for the regressors of interest were transformed into maps of *z* scores, coregistered with structural maps, spatially normalized by warping to Talairach space, and resampled as 2 mm^3^ voxels. Whole-brain voxelwise statistical thresholds were set to *p* < 0.001, uncorrected, as suggested for exploratory characterization (Cox et al., 2017). A minimum cluster size of 18 contiguous, face-to-face 2.9 mm^3^ voxels yielded a corrected whole-brain correction of *p* < 0.05 (after applying the 3dClustSim algorithm to a gray matter mask from AFNI version 18.0.25).

##### Data availability.

The preregistration for experiment 2 (https://osf.io/7pwnq) as well as relevant deidentified data and analytic code for both experiments (https://osf.io/yd8gn) are available on the Open Science Framework.

## Results

In both experiments, we initially tested whether subjects' choice behavior and stock indicators could forecast actual stock price dynamics. Next, we tested whether subjects' brain activity could forecast actual stock price dynamics—both before and after controlling for relevant behavioral and stock indicators. Finally, we conducted whole-brain analyses to confirm subjects' engagement and involvement of activity in predicted regions of interest in stock price movement forecasts.

### Choice behavior and stock indicators

Consistent with traditional finance theory (e.g., the efficient market hypothesis; Fama, 1970), we predicted that subjects' choices would not forecast stock price movements. Logistic regression analyses accordingly indicated that subjects' choice behavior could not significantly forecast the next-day stock price (Behavioral model; experiment 1: *z* = 1.60, *p* = 0.110; experiment 2: *z* = 0.51, *p* = 0.609; Table 1). Additionally, subjects behaved similarly across experiments (percentage of trials in which subjects chose to invest: experiment 1: mean = 54.89%, SD = 13.155%; experiment 2: mean = 53.45%, SD = 13.04%). Furthermore, subjects appeared to be similarly engaged across both experiments, since regression analyses predicting choice based on block number indicated that subjects' choices did not change over time (i.e., behavior did not differ among all 14 10-trial blocks: experiment 1: *t*_(441)_ = –1.23, β = –0.25, *p* = 0.221; experiment 2: *t*_(506)_ = 0.91, β = 0.020, *p* = 0.336).

**Table 1.** Logistic regression models forecasting aggregate stock price dynamics (experiment 1)

	Market model	Behavioral model	Neural model	Combined model
Intercept	1.157 (0.470)*	−1.041 (0.723)	−0.046 (0.195)	−0.135 (1.041)
Slope	−1.895 (1.689)			−2.943 (1.845)
Volatility	−0.055 (0.033)			−0.035 (0.036)
Previous trial	−0.954 (0.364)**			−1.262 (0.464)**
Choice		2.060 (1.291)		1.980 (1.519)
**NAcc activity**			6.227 (2.827)*	8.892 (4.151)*
NAcc*Prv Trial				−2.513 (7.136)
AIns activity			−2.610 (2.658)	5.038 (4.099)
AIns*Prv **trial**				−12.748 (6.101)*
MPFC activity			−0.659 (1.932)	−0.939 (3.216)
MPFC*Prv trial				−1.656 (4.622)
*R*^2^ ^‡^	0.057	0.014	0.030	0.136
χ^2^ model	10.834*	2.612	5.554	25.596**
AIC	185.438	189.659	190.717	184.675

Statistics are coefficients with SEMs in parentheses. Predicted associations in bold.

**p < 0.01;

*p < 0.05.

^‡^*R*^2^ is McFadden's pseudo-*R*^2^.

Another logistic regression analysis including stock indicators as predictors (i.e., the Market model with stock slope, volatility, and price movement on the previous day as fixed effects) revealed that the stock price direction of the previous day inversely forecast the stock price direction of the next day in experiment 1 (Market model; *z* = –2.62, *p* < 0.009; Table 1). This negative autocorrelation in stock prices may have provided subjects with information to aid their predictions. Thus, we pseudorandomly selected a set of stocks for experiment 2 to remove the potential confound of daily autocorrelation in prices (Market model; *z* = –0.60, *p* = 0.548; Table 2; see Materials and Methods) and thus support more robust verification of the generalizability of findings from experiment 1.

**Table 2.** Logistic regression models forecasting aggregate stock price dynamics (experiment 2)

	Market model	Behavioral model	Neural model	Combined model
(Intercept)	0.525 (0.494)	−0.275 (0.727)	0.122 (0.184)	0.365 (0.951)
Slope	−1.397 (1.725)			−1.634 (1.791)
Volatility	−0.028 (0.033)			−0.030 (0.035)
Previous trial	−0.207 (0.344)			−0.740 (0.412)
Choice		0.678 (1.324)		0.944 (1.517)
**NAcc activity**			**0.146 (2.852)**	**−0.625 (4.332)**
NAcc*Prv Trial				1.552 (6.061)
AIns activity			−0.996 (3.004)	6.143 (4.433)
AIns*Prv trial				−15.032 (6.534)*
MPFC activity			2.122 (1.779)	2.899 (2.714)
MPFC*Prv trial				−0.973 (3.701)
*R*^2‡^	0.008	0.001	0.010	0.058
χ^2^ model	1.670	0.263	1.947	11.099
AIC	197.378	194.785	197.101	201.949

Statistics are coefficients with SEMs in parentheses. Predicted associations in bold.

*p < 0.05.

^‡^*R*^2^ is McFadden's pseudo-*R*^2^.

### Brain activity

#### Volume of interest analyses: forecasting stock price dynamics

To test the critical hypothesis that brain activity could forecast stock price dynamics, further logistic regression analyses forecast next-day stock price movements using neural data alone (Neural model), as well as after combining neural variables with choice behavior and stock indicators (Combined model).

In experiment 1, the Neural model indicated that average NAcc activity positively forecast next-day stock price (*z* = 2.20, *p* = 0.028; Table 1). The Combined model indicated that prior price movement (*z* = –2.72, *p* = 0.007), NAcc activity (*z* = 2.14, *p* = 0.032), and the interaction of prior price movement with AIns activity (*z* = –2.09, *p* = 0.037) significantly forecast next-day stock price (Combined model; Table 1). This interaction also remained significant when including only AIns neural activity, prior price movement, and their interaction in a reduced model (*z* = –2.47, *p* = 0.013). Direct model comparisons indicated that the Combined model forecast stock price movements better than the Market model (χ^2^ = 14.76, *p* = 0.039), the Behavioral model (χ^2^ = 22.98, *p* = 0.006), and the Neural model (χ^2^ = 20.04, *p* = 0.005).

**Figure 2.**
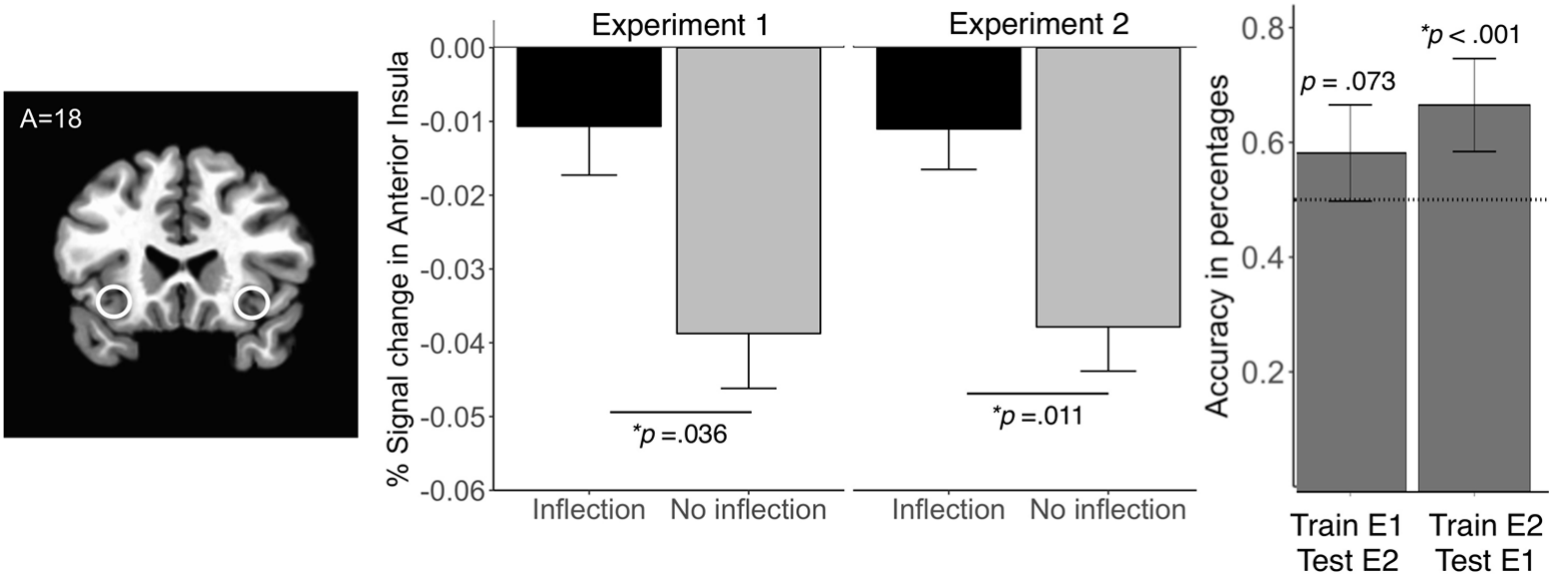
Anterior insula activity forecasts stock price inflections. Left, AIns VOIs; middle, AIns VOI activity is higher in trials involving an inflection (i.e., stock price decreases after a previous increase or increases after a previous decrease). Error bars depict SEM. *N*_exp 1_ = 34; N_exp 2_ = 39. Right, The interaction of AIns activity by previous stock price movement classifies out-of-sample stock price movement. First (second) bar depicts the accuracy of a reduced model trained on AIns activity, previous stock price movement, and their interaction in experiment 1 (2), and tested on experiment 2 (1). Dotted line indicates chance performance. Error bars depict 95% confidence intervals. *N*_exp 1_ = 34, N_exp 2_ = 39. exp, Experiment.

To decompose the interaction of price movement and AIns activity, we conducted *post hoc t* tests comparing AIns activity for price inflections (i.e., price decreased following an increase or vice versa) versus noninflections (i.e., price increased following an increase or vice versa). Generally, AIns activity forecast price inflections versus noninflections (mean_inflection_ = –0.011, SD_inflection_ = 0.080; vs mean_noninflection_ = –0.039, SD_noninflection_ = 0.070; *t*_(120)_ = 2.12, *p* = 0.036; Fig. 2). More specifically, AIns activity forecast price decreases that followed increases rather than price decreases that followed decreases (mean_increase→decrease_ = –0.003, SD_increase→decrease_ = 0.059; vs M_decrease→decrease_ = –0.044, SD_decrease→decrease_ = 0.060; *t*_(53)_ = 2.70, *p* = 0.009). Although both NAcc and AIns activity forecast stock price dynamics (in the Combined model) when choice did not (in the Behavioral model), the significant autocorrelation in the stock prices in this experiment (in the Market model) motivated a preregistered second experiment which included stock prices without autocorrelation.

Unlike experiment 1, the Neural model in experiment 2 did not show significant associations of NAcc activity with stock price dynamics (Neural model: NAcc, *z* = 0.05, *p* = 0.959; Table 2). Similar to experiment 1, though, the Combined model (which included choice, stock indicators, and neural data as predictors) in experiment 2 continued to show a significant interaction of prior price movement with AIns activity (*z* = –2.30, *p* = 0.021; Table 2). This interaction again remained significant when including only AIns neural activity, prior price movement, and their interaction in a reduced model (*z* = –2.39, *p* = 0.017). Direct model comparisons, however, did not reveal that the Combined model significantly outperformed the other models.

As in experiment 1, AIns activity generally forecast price inflections versus noninflections (mean_inflection_ = –0.011, SD_inflection_ = 0.065; mean_no inflection_ = –0.037, SD_no inflection_ = 0.058; *t*_(136)_ = 2.59, *p* = 0.011; Fig. 2). Again, AIns activity specifically forecast price decreases that followed increases versus decreases that followed decreases (mean_increase→decrease_ = –0.011, SD_increase→decrease_ = 0.055; mean_decrease→decrease_ = –0.041, SD_decrease→decrease_ = 0.053; *t*_(62)_ = 2.31, *p* = 0.024). Although the Combined model appeared to account for the most variance in experiment 2 (i.e., larger pseudo-*R*^2^), the fit was less robust than other models (i.e., larger AIC), suggesting potential overfitting. Therefore, we sought to more robustly test the generalizability of the interaction of AIns activity with previous trial price movement with classifier tests.

#### Classifier tests of generalization

A classifier trained on data from the Combined model of experiment 1 forecast stock price movement in data from experiment 2 with 59.42% accuracy (95% CI = ±8.19%), which exceeded chance (or 50% accuracy; *p* = 0.033, binomial test). A reduced version of this classifier trained on a model only including AIns neural activity, prior price movement, and their interaction in data from experiment 1 showed that this interaction continued to forecast the stock prices of experiment 2 with 57.97% accuracy (95% CI = ±8.23%), which exceeded chance at a trend level (*p* = 0.073, binomial test; Fig. 2). Further, classifiers trained on randomized stock prices from experiment 1 could not forecast next-day stock prices in experiment 2 (Combined model: *t*_(499)_ = 1.39, *p* = 0.165; reduced model including only AIns neural activity, prior price movement, and their interaction: *t*_(499)_ = −1.134, *p* = 0.257).

Conversely, a classifier trained on data from the Combined model of experiment 2 forecast stock price movement in data from experiment 1 with 63.97% accuracy (95% CI = ±8.06%), which exceeded chance (*p* = 0.001, binomial test). A reduced version of this classifier trained only on AIns neural activity, prior price movement, and their interaction in experiment 2 continued to forecast stock prices from experiment 1 with 66.18% accuracy (95% CI = ±7.95%), which exceeded chance (*p* < 0.001, binomial test; Fig. 2). Again, classifiers trained on randomized stock prices from experiment 2 could not forecast next-day stock prices in experiment 1 (Combined model: *t*_(499)_ = −0.292, *p* = 0.77; reduced model including only AIns neural activity, prior price movement, and their interaction: *t*_(499)_ = 0.758, *p* = 0.449). Together, these findings suggest that the interaction of group AIns activity with the stock price of the previous day contains information capable of forecasting next-day stock price movement, even out-of-sample.

#### Whole-brain confirmatory analyses

A first whole-brain analysis confirmed predicted responses to incentive outcomes and task engagement. As predicted, NAcc activity increased both in response to gains (i.e., price increases after choosing to invest) and to avoided losses (i.e., counterfactual price decreases after choosing not to invest). Conversely, NAcc activity decreased both in response to losses (i.e., price decreases after choosing to invest) and to missed gains (i.e., counterfactual price increases after choosing not to invest; Table 3).

**Table 3.** Whole-brain responses to actual and counterfactual gain versus loss outcomes

Gain versus loss outcomes						Counterfactual gain versus loss outcomes					
Region	*x*	*y*	*z*	Peak *z*	#V	Region	*x*	*y*	*z*	Peak *z*	#V
Experiment 1						Experiment 1					
**L NAcc**	**−10**	**7**	**−3**	**6.23**	**339**	**L NAcc**	**−13**	**10**	**−6**	**5.88**	**100**
**R NAcc**	**13**	**10**	**−6**	**6.12**	**335**	**R NAcc**	**13**	**12**	**−3**	**5.08**	**94**
L angular gyrus	−45	−57	35	4.55	300	R putamen	30	−8	3	4.69	85
L sup frontal gyrus	−19	21	46	4.98	206	R lingual gyrus	22	−89	−3	4.47	62
L inf frontal gyrus	−45	33	8	5.22	192	R supramarginal gyrus	54	−40	35	4.33	43
L cingulate gyrus	−4	−37	38	4.97	182	R mid frontal gyrus	30	33	35	4.03	35
L med frontal gyrus	−19	−8	49	4.09	73	R anterior cingulate	1	42	14	4.06	30
L inf temp gyrus	−48	−19	−18	4.48	64	R precentral gyrus	48	10	6	3.82	18
R inf temp gyrus	56	−28	−15	4.23	53	Experiment 2
L ant cingulate	−2	42	12	4.10	51	**R NAcc**	**13**	**10**	**−9**	**5.91**	**398**
R angular gyrus	36	−60	32	4.20	48	R inf temp gyrus	39	−69	−0	5.44	243
R sup frontal gyrus	25	33	43	4.49	45	L mid occipital gyrus	−33	−74	−0	5.21	181
R inf parietal lobule	48	−46	43	3.97	42	R inf parietal lobule	36	−43	43	4.07	152
L med frontal gyrus	−2	27	38	3.75	39	**L NAcc**	**−16**	**10**	**−6**	**5.69**	**113**
L inf frontal Gyrus	−22	24	−12	4.59	25	Right precentral gyrus	39	−2	26	4.43	91
R cerebellar tonsil	42	−54	−38	3.87	24	L cerebellum	−25	−63	−26	4.70	53
R mid frontal gyrus	28	53	3	3.73	22	R fusiform gyrus	45	−51	−9	4.25	31
Experiment 2						R mid frontal gyrus	36	−2	55	3.82	31
**R (+L) NAcc**	**16**	**7**	**−3**	**7.51**	**14381**	L precuneus	−22	−51	46	4.10	28
R cerebellar tonsil	42	−54	−41	6.00	214	L supramarginal gyrus	−39	−37	38	3.93	26
R sup temp gyrus	56	−57	23	5.30	163	R mid frontal gyrus	36	39	6	3.70	19
R parahippocampal gyrus	25	−31	−6	4.53	51	R precentral gyrus	45	21	35	3.94	19
R mid frontal gyrus	30	30	29	4.03	46						
R culmen	25	−31	−20	4.56	35						

Threshold: *z* = 3.29, *p* < 0.001, uncorrected. Cluster, minimum 18 voxels; voxel size = 2.9 mm^3^; Talairach coordinates: L, left; R, right; mid, middle; temp, temporal; sup, superior; inf, inferior; #V, number of voxels. Predicted associations in bold.

**Figure 3.**
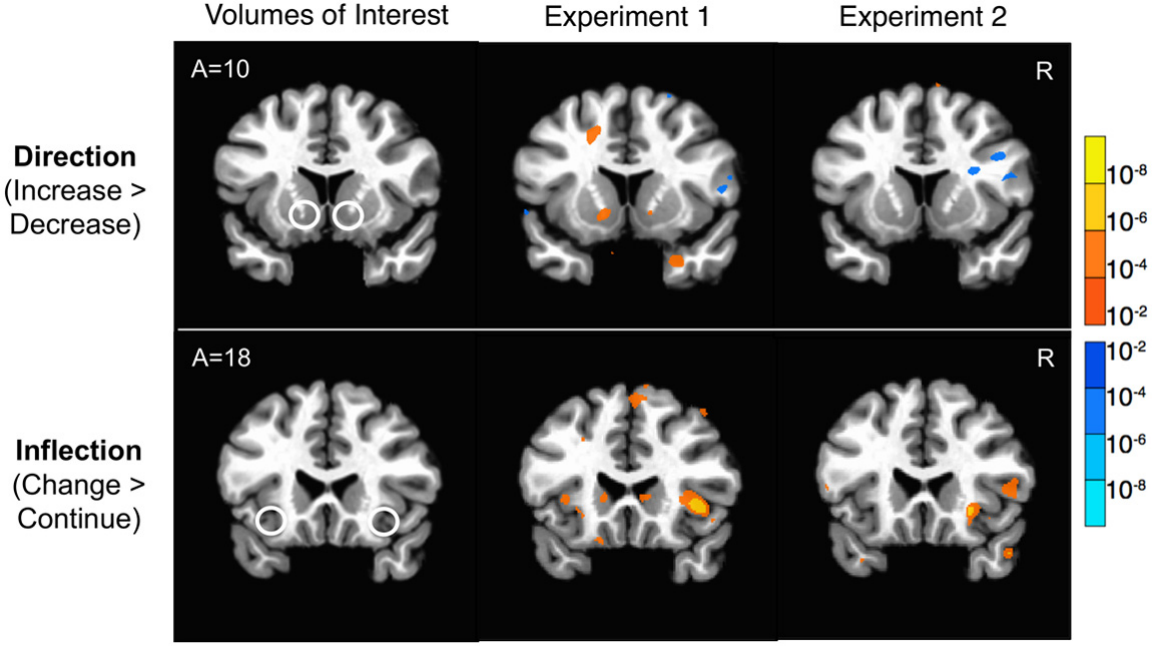
Whole-brain confirmation that activity in predicted regions forecasts stock price direction and inflection. Left, White circles indicate VOIs. Top, Stock price direction: NAcc activity forecast stock price direction in experiment 1 (middle), but not experiment 2 (right). Bottom, Stock price inflection: AIns activity forecast stock price inflection in experiments 1 (middle) and 2 (right). Whole-brain analysis, *N*_exp 1_=34, *N*_exp 2_=39. Statistical overlay thresholded at *p* = 0.01, uncorrected for display. exp, Experiment.

A second whole-brain analysis confirmed the selection of VOIs whose activity forecast stock price direction and inflection (Fig. 3, Table 4). In experiment 1, whole-brain analyses of neural activity associated with subsequent stock price direction (i.e., when the price increases after increases or decreases after decreases) suggested that left NAcc activity forecast stock price increases, but only at a predicted small-volume threshold (i.e., 2 voxels at *p* < 0.005, uncorrected; 7 voxels at *p* < 0.01, uncorrected). Whole-brain analyses of neural activity associated with stock price inflection (i.e., when the price decreased after a previous increase or increased after a previous decrease) indicated that increased right AIns, bilateral dorsal striatum, occipital cortex, and dorsal MPFC activity preceded stock price movement inflections (*p* < 0.001, uncorrected). In experiment 2, while left NAcc activity did not forecast stock price direction [instead, activity in the occipital cortex, posterior cingulate cortex, and the MPFC (four voxels) forecast stock price direction at *p* < 0.001, uncorrected], increased right AIns activity still forecast stock price inflections (*p* < 0.001, uncorrected; Fig. 3).

**Table 4.** Whole-brain activity forecasting stock price direction (price continues) and inflection (i.e., price changes)

Stock price direction						Stock price inflection					
Region	*x*	*y*	*z*	Peak *z*	#V	Region	*x*	*y*	*z*	Peak *z*	#V
Experiment 1						Experiment 1					
L cuneus	−10	−95	8	3.85	31	R precuneus	25	−66	32	5.80	632
R mid occipital gyrus	25	−86	0	−5.01	21	L sup occipital gyrus	−30	−72	29	5.28	519
Experiment 2						R medial frontal gyrus	1	33	40	4.31	105
L cingulate gyrus	−2	−46	35	4.19	74	L inf temporal gyrus	−57	−37	−18	5.02	69
R mid occipital gyrus	39	−74	3	−4.58	37	R pallidum	10	−2	3	4.54	66
L precuneus	−2	−66	26	3.57	22	L cuneus	−13	−74	12	4.46	64
L cerebellar lingual gyrus	−13	−83	−6	4.07	20	L pallidum	−13	1	3	4.31	37
						**R ant insula**	**39**	**18**	**0**	**4.64**	**29**
						L precuneus	−13	−74	43	5.05	28
						R thalamus	10	−14	14	4.82	26
						L thalamus	−7	−16	12	4.30	25
						L cerebellar declive	−30	−57	−12	4.43	21
						R cingulate gyrus	1	−34	26	4.59	21
						R cuneus	10	−69	14	3.90	19
						Experiment 2					
						**R ant insula**	**28**	**18**	**−3**	**3.97**	**22**

Whole-brain analysis: threshold *z* = 3.29, *p* < 0.001, uncorrected. Cluster, Minimum 18 voxels; voxel size, 2.9 mm^3^; Talairach coordinates: L, left; R, right; mid, Middle; temp, temporal; sup, superior; inf, inferior; #V=number of voxels. Predicted associations in bold.

## Discussion

In two neuroimaging experiments, we examined whether brain activity could forecast next-day movements in stock prices. Results indicated that group AIns activity could forecast stock price inflections (i.e., changes in price direction) across two different stock markets. Group NAcc activity could also forecast price direction (i.e., continuing price movement), but only in a market with autocorrelation in stock prices. Importantly, group choice behavior could not forecast stock prices, implying that the findings could not be attributed to learning over time or to correlated stock price histories. These findings suggest that neural activity associated with anticipatory affect can forecast aggregate choice—even in dynamic and competitive environments like stock markets. The results extend previous research using brain activity to predict risky choices of individuals, in which NAcc activity has been associated with positive arousal and risk-seeking choices, but AIns activity has been associated with general or negative arousal and risk-averse choices (Kuhnen and Knutson, 2005; Preuschoff et al., 2006; Lohrenz et al., 2007).

These findings are also consistent with a “partial scaling” account of aggregate choice, in which some components underlying individual choice generalize to forecast aggregate choice better than others, including subsequent behavior (Knutson and Genevsky, 2018). The partial scaling account lies between “total scaling” accounts in which individual choices simply add up to generate aggregate choice (e.g., expected value) and “no scaling” accounts in which individual choices yield no information about aggregate choice (e.g., the efficient market hypothesis; Fama, 1970). If no scaling accounts posit that choice behavior should not consistently forecast stock price movements, then, by extension, neither should its components. Yet, in both experiments, the interaction of group AIns activity with previous stock price movements forecast stock price inflections. Further, cross-validation analyses demonstrated that this neural marker generalized across markets (which varied in terms of subjects, stock identity, and price dates). Thus, these findings provide an initial demonstration that experimentally sampled AIns activity can forecast aggregate stock price dynamics.

While AIns activity forecast stock price inflections, it remains unclear which features of stock prices previously influenced AIns activity. Behavioral researchers have found that individuals can distinguish stock price sequences from randomized but otherwise similar sequences, but have not identified which stock features facilitate this distinction (Hasanhodzic et al., 2019). The present analyses suggested that AIns responses to conventional stock indicators (e.g., the direction of price movement on the previous day, the direction of slope, or the volatility of current stock price movements) could not forecast price inflections in a straightforward way. AIns activity might instead respond to more complex or even mutually exclusive dynamics in stock prices. Based on previous neuroimaging research implicating AIns activity in arousal and uncertainty (Critchley et al., 2001; Clark et al., 2014), various stock features that induce surprise or doubt might generally increase AIns activity. The present findings do not specify, however, exactly which input patterns induce the psychological uncertainty and associated neural activity that contributed to forecasts, a topic that remains ripe for further inquiry. The degree to which rapid and dynamic neural correlates of anticipatory affect are accessible to conscious report is also unclear, but deserves further targeted investigation.

Although medial prefrontal cortical activity often predicts individual choice, including financial investments (De Martino et al., 2013; Frydman et al., 2014), MPFC activity did not forecast aggregate stock price movements in these experiments. A partial scaling account posits that neural components related to anticipatory affect (e.g., the NAcc) lie lower in the brain and are more evolutionarily conserved, whereas components related to value integration (e.g., the MPFC) lie higher and nearer to behavioral output (Haber and Knutson, 2010). While neural activity related to anticipatory affect might generalize more broadly across people to forecast aggregate choice (Knutson and Genevsky, 2018), neural activity related to value integration might instead extend more narrowly within individuals across time to promote personal choice consistency (Camille et al., 2011).

Few studies have examined NAcc or AIns activity in the context of aggregate stock market events (Barton et al., 2014), although in one study, experimentally sampled NAcc activity tracked experimentally produced market bubble formation, and individuals who showed greater AIns activity tended to exit experimental market bubbles earlier and reap higher returns (Smith et al., 2014). With the exception of a single patient case study of NAcc dopamine release (Kishida et al., 2011), however, research has not yet used experimentally sampled brain activity to forecast actual stock price dynamics. Further, although several neuroforecasting studies have implicated NAcc activity in forecasting aggregate choice (Knutson and Genevsky, 2018), only one study of an Internet attention market (i.e., https://youtube.com) has implicated AIns activity in lower video engagement (Tong et al., 2020).

In the current experiments, AIns activity provided the most generalizable forecasts. The ability of AIns activity to forecast aggregate choice in this research may depend on the types of choices that predominate in stock markets in contrast to other markets. While previous research has primarily focused on markets involving purchases of goods, stock markets require investors to weigh uncertain gains (or “goods”) against uncertain losses (or “bads”). Outside the laboratory, forecasting stock price inflections (or reversals) may present a more formidable challenge than forecasting stock price direction (or momentum). Despite the practical challenges inherent in applying neuroimaging data to forecasts of stock price dynamics (e.g., the difficulty of sampling neural data immediately before price changes), neural measures may eventually yield valuable “hidden information” that is otherwise difficult to obtain (Ariely and Berns, 2010).

This research features a number of novel strengths, including the use of actual stock price data, direct quantitative comparisons of qualitatively distinct predictors (e.g., stock indicators, behavior, and neural activity), out-of-sample cross-validation, and a replication experiment that controlled for temporal structure in stock prices. Limitations, however, include necessarily constrained sets of stock scenarios (necessitated by time limits typical of scanning experiments), simplified presentation of information (e.g., distilled from more conventional but variable trading information interfaces and timescales), and use of historical (though recent) data. All of these variables deserve systematic exploration in future research. Many interesting questions also remain with respect to individual differences (e.g., whose behavior and brain activity best forecast stock price movement), generalizability to more complex trading environments, potential influence of prior trading experience, and conditions under which behavior adds value to neural forecasts.

Overall, this research extends neuroeconomic theory by implying that brain activity associated with anticipatory affect can forecast aggregate choice—even in complex markets involving dynamic strategic interactions between actors (Kirman, 1992). Additionally, the current findings challenge traditional theoretical accounts that imply elements of choice cannot inform financial forecasts (Fama, 1970) by demonstrating that previously hidden neural activity might provide uniquely valuable information about stock price dynamics.
